# Rare cystic lymphangioma in the chest wall of an adult patient: A case report and comprehensive review of the literature

**DOI:** 10.1111/1759-7714.13659

**Published:** 2020-09-28

**Authors:** Shuai Song, Dong Chang, Hao Li, Bowen Li, Kaikai Xu, Chunquan Liu, Yong Cui

**Affiliations:** ^1^ Department of Thoracic Surgery, Beijing Friendship Hospital Capital Medical University Beijing China

**Keywords:** Chest wall, cystic lymphangioma, surgical resection

## Abstract

Lymphangiomatosis is a rare, benign, hyperproliferative hamartoma composed of dilated lymphatic vessels. Cystic lymphangioma (CL) in the chest wall in an adult patient is rare, but we focus on this type of patient in our present case study. A 54‐year‐old female patient with a painless mass in her chest wall went without treatment for two years following diagnosis. After consenting to treatment, Doppler color flow imaging (DCFI), chest CT, and MRI revealed a cystic lesion with multiple thin septula in the left chest. Surgical resection was performed, and histopathological examination identified a cystic lymphangioma. The patient did not experience recurrence during the follow‐up period.

## Graphical abstract

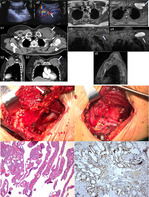

Cystic lymphangioma is a rare disease occurring infrequently in the chest wall of adult patients. MRI is useful for diagnosis and surgical resection is recommended as the main treatment.

## Introduction

Cystic lymphangioma (CL) is a rare disease and considered a congenital malformation characterized by the proliferation of lymphatic vessels, resulting from failure to establish communication between primary lymphatic sacs and the venous system.^1^ Although the incidence of adults with CL is very low, diagnosis and treatment strategy are always of great concern to thoracic surgeons. Most cases of CL are congenital and the incidence in the pediatric population is approximately one new case per 12 000 births.^2^ In some rare conditions, it can be an acquired malformation resulting from injury, inflammation, or fibrosis.^3^ CL of the chest wall is extremely rare in adults and there are very few previous reports of its occurrence. The chest wall is a rare location for presentation of CL and this occurrence was first described by Omell *et al*. in 1973.^4^ Here, we present an adult patient with lymphangioma in which we review the process of diagnosis and treatment, and systematically research the features of CL in adults.

## Case report

A tumor had been identified in a 54‐year‐old female patient in the left chest wall two years previously. The tumor was about 4 cm in diameter and no pain had been experienced by the patient. Chest ultrasonography revealed an irregularly shaped hypoechoic cystic mass with an obscure boundary. The patient had refused medical treatment because at the time no uncomfortable feelings were being experienced; however, the mass gradually increased in size. Feelings of discomfort arose for more than six months, and continued to become more aggravating. The patient was then referred to our hospital. Physical examination detected a hemispherical, painful, pliable, subcutaneous lump below the left clavicle, with a diameter of about 4 cm, unclear boundaries, and poor mobility. No ecchymosis was found in the overlying skin. Transillumination was not feasible because of the location. At this point, the patient accepted ultrasonography, including Doppler color flow imaging (DCFI), which revealed a cystic lesion with multiple thin septula. No blood flow was detected on echodoppler imaging. Chest computerized tomography (CT) scan revealed an irregular, low density mass, located between the pectoralis major and pectoralis minor muscles. With dimensions of 3.2 × 6.2 cm, it was closely adjacent to the bony chest wall without extension into the thoracic cavity. CT value was 16 HU. There was no enhancement on CT scanning. Magnetic resonance imaging (MRI) was recommended and an irregular shaped mass was found; imaging revealed low density in T1WI and high density in T2WI + FS. Enhanced scanning detected an enhanced capsule wall, with no invasion of bone or soft tissue.

According to the results, a diagnosis of a vascular tumor was made. No contraindications were found, and it was decided to surgically resect the tumor. The patient was placed under general endotracheal anesthesia and a skin incision under the surface of the lesion was made. Subcutaneous fat tissue was separated from the myofibers of the pectoralis major muscle. A cystic mass with high attenuation was found, approximately 8 × 6 × 3 cm in size, containing crimson bloody fluid. It was adherent to surrounding soft tissue and rib, and partially blocking the subclavian vein. The mass was excised completely without bleeding and the patient recovered quickly without complications. Histopathological examination documented the mass as an irregular, thickened, fibrous capsule covered by flat endothelial cells with muscle fibers and fat tissues located on the outer surface. Immunohistochemical examination revealed CD3+, CD34+, and D2‐40+. A diagnosis of cystic lymphangioma was made in accordance with the pathological features and immunohistochemical results. The patient had no evidence of recurrence during follow‐up in the following 10 months.

## Discussion

According to its location, CL of the chest is divided into mediastinal, pulmonary, and chest wall lymphangioma. Chest wall CL is rare; furthermore it usually occurs in children and young patients^2^ and there are few reports of adult patients with CL. CL is diagnosed at birth in 50% of all cases and diagnosed in 90% of all cases by the age of two years old. While there are some cases where diagnosis has been delayed, the mean age of diagnosis is three years old.^5^, ^6^ In the case reported here, the patient was an adult female, with nonspecific symptoms and manifestations.

Ultrasonography and CT substantially contribute to the diagnostic approach for chest wall cystic lymphangioma. During ultrasonographic examination, chest wall lymphangioma appears as a multicystic shadow of a mass with water‐like density that is closely connected to the chest wall.^7^ The shape is regular, and the borders are clear. The muscle and fat structure in the adjacent part of the mass are clear. Lymphoma of the posterior chest wall is more common, which may be related to the more relevant distribution of posterior lymphatic vessels in the chest than in other regions. CT scan shows that lymphangioma has relatively uniform low density, which reflects the characteristics of lymphatic lumen containing fluid. In the case reported here, the enhanced scan revealed only slight enhancement of the margins and separation in the lesion.^8^ After the delayed scan, the main body of the lesion still lacked enhancement, reflecting the lack of histological features of the disease. In recent years, MRI has been commonly performed for the diagnosis of chest disease.^9^ MRI has made it easier to distinguish between chest mass diseases and provide more detail on airway pressure, thoracic expansion, and internal organ or bone lesions. In our case, MRI showed an irregularly shaped mass, revealed low density in T1WI and high density in T2WI + FS. Enhanced scanning found an enhanced capsule wall and determined that no bony or soft tissue had been invaded. MRI therefore plays an important role in distinguishing a diagnosis from other chest tumors and cysts.

Histopathological examination and immunohistochemical techniques are the most useful tools for diagnosing CL. Tissue section observation was done on a multicavity tumor lumen that had no red blood cells except for individual small blood vessels. Observation through ultra‐thin section projection electron microscopy showed that there were vesicles of different sizes in the tumor cytoplasm. These vesicles further developed and appeared in the cells to merge to form a typical endothelium‐like cell‐lined lumen.^8^ Endothelium‐like cells can constitute the wall of a single lumen or they can participate in the formation of multiple lumen sacs, indicating that the tumor may be caused by lymphatic endothelium at different developmental stages. The cells form a misshapen lymphatic vessel and sac cavity. D2‐40 and lymphatic vessel endothelial receptor‐1 (LYVE‐1) are the immunohistochemistry markers for diagnosis.^10^ In our case, the diagnosis was confirmed by histopathological examination.

Surgical resection is necessary for treatment, as it is important to perform complete resection, including the circumferential tissues, in order to prevent recurrence. When surgical intervention is not feasible, systemic chemotherapy or the administration of interferon‐α are recommended,^11^ however, prognosis is poor in follow‐up.^12^ In addition, some new treatments have recently been reported; intralesional sclerotherapy (IS) is considered as an alternative treatment for CL.^13^ Unfortunately, complications have been reported resulting in difficulties in the application of treatment and results are still controversial.^14^ The administration of propranolol is also considered useful for the treatment of CL,^15^ but long‐term follow‐up is necessary.

After surgery, the patient in our study recovered well and there was no evidence of recurrence during follow‐up in the next 10 months. However, there have been studies which found that CL patients relapsed after surgery. Flanagan *et al*. reported a case of relapse seven years postoperatively,^16^ while Lee *et al*. reported a case of chest wall cystic lymphangioma that relapsed 19 years after surgical removal.^5^ We are of the opinion that our patient should attend regular follow‐up checks for an extended period of time.

We conclude that cystic lymphangioma of the chest wall does not only occur in children and younger patients, and whilst cases of CL in adult patients are rare, they are found. In the process of disease diagnosis and treatment options for CL, MRI is extremely useful for diagnosis and surgical resection is recommended as the main form of treatment.

## Disclosure

The authors declare no potential conflicts of interest. All authors certify that they have no affiliations with, or involvement in, any organization or entity with any financial interest (such as honoraria, participation in speakers' bureaus, membership, employment, consultancies, stock ownership, or other equity interest, expert testimony or patent‐licensing arrangements), or nonfinancial interest in the subject matter or materials discussed in this manuscript.
